# Acupuncture and oxytocinergic system: The promising treatment for autism

**DOI:** 10.1515/tnsci-2021-0011

**Published:** 2021-02-16

**Authors:** Tangfeng Su, Lei Pei

**Affiliations:** Department of Pediatrics, Tongji Hospital, Tongji Medical College, Huazhong University of Science and Technology, Wuhan, Hubei, 430030, China; Department of Neurobiology, School of Basic Medicine, Tongji Medical College, Huazhong University of Science and Technology, Wuhan, 430030, China; Department of Anesthesiology, Washington University in Saint Louis School of Medicine, Saint Louis, MO, 63110, USA; The Institute for Brain Research (IBR), Collaborative Innovation Center for Brain Science, Huazhong University of Science and Technology, Wuhan, China

**Keywords:** autism spectrum disorder, oxytocin, endocannabinoid, serotonin, dopamine, nucleus accumbens, ventral tegmental area

## Abstract

Autism spectrum disorder (ASD) is a group of heterogeneous neurodevelopmental disorders characterized by impairments activities without efficient pharmacological therapies in social interaction, speech and stereotypic patterns. Clinical studies have shown the efficacy of acupuncture as an alternative therapy for autism. The effectiveness of acupuncture as an alternative treatment for autism has been demonstrated through clinical trials. However, the molecular mechanisms that underlie these effects remain unclear. Due to its profound pro-social, anxiolytic, stress management effects, and its potential use for the treatment of psychiatric disorders associated with altered socioemotional competence, oxytocin (OT) released from the hypothalamus has attracted considerable interest. In the past decade, a number of clinical and animal studies have shown that OT administration effectively reduces core symptoms of ASD, especially social behavior deficits. Recently, the endocannabinoid system has emerged as a promising target for the treatment of autism. OT was found to facilitate the endocannabinoid-mediated social reward processes in the nucleus accumbens of the mouse brain. Furthermore, serotonin and dopamine are involved in the reward response mediated by OT. In view of these findings, we conclude that acupuncture may produce therapeutic effects on autism by triggering the hypothalamic oxytocin system, which in turn activates the release of neurotransmitters such as endocannabinoids, dopamine and serotonin. This would be a valuable guide for further research on the mechanism of treatment of autism with acupuncture.

Autism spectrum disorder (ASD) is a developmental disability, typically manifested as difficulties in social interaction, repetitive behaviors and deficiency in language communication, which seriously affects the quality of life of patients. So far, there are no effective medicines for the treatment of ASD. However, studies indicate that early intervention can improve children’s development. The cause of ASD has not been fully determined, but cumulative evidence has shown that the onset of ASD is the result of multifactor interactions, including gene mutation, environmental contamination, and abnormal development of central nervous system (CNS) [1,2]. In terms of the nervous system, the pathogenesis of ASD is closely associated with damaged neurotransmitter systems in the nervous system, such as serotonin (5-HT), norepinephrine, dopamine, and pineal–hypothalamic–pituitary–adrenal axis dysfunction, and related neuropeptides and hormones [3,4,5].

Peñagarikano et al. demonstrated that oxytocin (OT) significantly improves social behavior in a mouse model of autism, and that this effect can be sustained over a longer period of time if treated early [6]. Recently, a research group from Hamamatsu Medical University in Japan announced that they have shown a tendency to improve symptoms in people with ASD after injecting with OT (a trial of TTA-121 on autism spectrum disorder). It is known that OT is mainly produced by the giant cells in the supraoptic nucleus (SON) and paraventricular nucleus (PVN) of the hypothalamus, then transported through the nerve fibers of the hypothalamic pituitary axis to the posterior pituitary gland, and finally released into the blood, acting on various organs throughout the body, and finally exerts its important biological functions [7]. In women, OT can trigger uterine contractions in childbirth, stimulate milk secretion, and create contact between the mother and the child through affection. In addition, OT can also reduce the levels of corticosterone and other stress hormones in the body, and lower the blood pressure [8]. Overall, OT is critical for enhancing social behavior deficits associated with ASD, establishing relationships and engaging in social interaction, the abnormal concentration, or functions of OT is closely linked to autism pathogenesis.

## 1 OT deficiency is associated with social deficits in autism

OT is a highly conserved 9-peptide that is synthesized by neuronal cells in the PVN and SON of the hypothalamus and then released into the blood through the axonal endings of the posterior lobe of the neurohypophysis. Endogenous OT receptors (OTRs) have a broad distribution in the brain, particularly in the entire limbic system. The central neurotransmitter-like action of OT has been predominantly shown to modulate cognitive effects, regulating the development and maintenance of complex social and bonding behaviors.

Accumulative evidence indicates that OT plays a putative role in social communication, and the lack or under-use of OT may be connected to disorders related to social communication. Decreased OT and its receptor may lead to low social activity and autism-like behaviors, and this shift in the OT system is commonly observed in patients with ASD. In 1998, Modahl et al. first reported that OT deficiency in autistic children could be the cause of social disorders. They found that plasma OT levels were significantly lower in autistic children than in normal children of the same age, and the decrease in OT level was significantly linked to the degree of social communication impairment in autistic children. They also found that plasma OT levels did not increase with age in autistic children, as normal children did [9]. In addition, social deficits and reduced OT immunostaining have been reported in several models of autism, such as the developmental hyperserotonemia (DHS) model [10], the Cntnap2 mouse model [6], the prenatal valproic acid (VPA) rats model [11], and Shank3b^−/−^ mutant mice model [12]. In the Cntnap2 and VPA model, exogenous OT administration in early life was sufficient to rescue decreased OT immunoreactivity as well as social functioning, indicating that a crucial developmental time window could be required for optimal treatment [6,11]. These results indicate that a key cause to the development of autism could be the reduction or dysfunction of OT.

During long labors, the desensitization of OTR with long infusion periods of Pitocin and high infusion rates may be followed by down-regulation of OTR, which may be a cause that contributes to autism in offspring [13,14,15]. Furthermore, studies in OT [16] or OTR [17] gene knockout mice have shown abnormal social behaviors and ASD-like traits related social memory deficits. Animal experiments and clinical studies also have shown that intranasal or peripheral OT injection can obviously promote social interaction [6,18], but the therapeutic mechanism remains unclear. The possible mechanism could be linked to the excitatory–inhibitory shift of OT-mediated neuroprotective GABA during the perinatal period [19]. In ASD rodent models, the early birth GABA excitatory–inhibitory shift is disrupted, leading to greater excitatory GABA transmission and the followed autism-like behavior. The number of OT-immunoresponsive (OT-ir) cells in the SON of neonatal VPA autism rats was significantly lower than that of the control group. Early postnatal OT treatment (subcutaneous injection for 7 days from 24 h after birth) has a long-term therapeutic effect and can relieve social disorders and repetitive behavior before puberty. These behavioral changes are accompanied by an increase in the number of OT-ir cells [11].

## 2 OT deficiency is attributed to the stereotyped repetitive behaviors in autism

Another main characteristic of autism is repetitive and stereotypic behavior, which often occurs in other mental and psychological disorders, such as Angelman’s syndrome, Tourette’s syndrome, and obsessive-compulsive disorder. The OT system has been linked to the regulation of repetitive behavior in both animal models and human patients. For example, the OTR knockout mice showed high frequency of repetitive stereotyped behavior, such as increased self-grooming [20] and marble burying [21]. After OT treatment, these repetitive and stereotyped behaviors were substantially reduced [21,22].

In children with ASD, the plasma OT level was considerably lower than that in normal children. The correlation between OT and the repetitive stereotyped behaviors was found to be significant. Lower OT levels are often accompanied by more severe symptoms of stereotyped behavior in ASD children [23]. Furthermore, clinical trials have shown that OT administration effectively mitigated repetitive stereotyped behavior and improved comprehension of affective speech [24,25].

## 3 Acupuncture can cause prosocial effects of oxytocin

Although human intranasal OT studies illustrate a new hope for ASD, it should be noted that these studies had small sample sizes and some research identified relatively poor replicability, suggesting that intranasal OT studies are generally low statistical power [26]. Exogenous intranasal OT administration may also cause side effects such as nasal irritation, light headedness, drowsiness, and/or headache [27]. Bypassing the blood-brain barrier during exogenous pharmacological intervention remains a problem that needs to be tackled for a long time. Some research has turned to promoting endogenous oxytocin signaling [28].

Acupuncture is an important part of traditional Chinese medicine, which has been widely used as an alternative therapy for a variety of diseases. Acupuncture at specific acupoints regulates the neuro–immuno–endocrine network [29]. The hypothalamus is the key element of the hypothalamic–pituitary–adrenal cortex (HPA) axis, which perceives a multitude of signaling including stressful stimulations and acupuncture signals. It has been confirmed that acupuncture at specific acupoints specifically activates stress reaction neurons (SRNs) in PVN and influences the CNS through the HPA axis [30]. Several lines of evidence indicate that acupuncture treatment can improve the social and emotional responses of autistic patients characterized by impaired social behavior [31,32,33,34]. Studies have shown that acupuncture or electroacupuncture (EA) stimulation with unique frequency can promote the release of specific neurochemicals in the CNS and produce noticeable physiological effects [35]. For example, rats exposed to 30 min of 2 Hz EA treatment showed significant increase of the OT levels in plasma and in the cerebrospinal fluid (CSF) [36]. In addition, 10–20 Hz EA treatment can upregulate OT level in the SON of the hypothalamus in rats [37].

The damage of central OT system was reported for the first time in VPA autism rat model [11]. Compared with the control group, the OT mRNA level and peptide concentration in hypothalamus of the male and female VPA rats were much lower than that of the control animals, and the level of OT peptide in CSF remarkably decreased. Zhang et al. found that EA stimulation (2/15 Hz dense-disperse pulses wave) led to a selective increase in the expression of OT and arginine–vasopressin (AVP) in the hypothalamus. Furthermore, 2/15 Hz EA treatment increased the social interaction time in low socially interacting (LSI) rats. In addition, single-sequence EA stimulation can up-regulate the mRNA levels of hypothalamic OT and AVP. Similarly, repeated sequence EA stimulation can up-regulate the levels of OT and AVP mRNA and promote protein expression in SON, accompanied by a significant increase in serum AVP and enhanced sociability. The social behavior of rats was positively correlated with the mRNA levels of OT and AVP in the hypothalamus, suggesting that the socialization-promoting response observed after EA may be at least partially mediated by the upregulation of OT and AVP in the brain [38].

Based on the above studies, acupuncture may ameliorate the behavioral symptoms of autism by up-regulating the level of OT and AVP in the hypothalamus. Transcutaneous electrical acupoint stimulation (TEAS) is a non-invasive acupuncture-like technique, which is easy to apply in young children. The physiological effects of TEAS are similar to that of EA on analgesia [39] and other diseases [40]. Acupuncture intervention at an early age or during developmental critical periods may be beneficial to the facilitation of AIDRR (Activate, Integrate, Discriminate, Respond, Reward) neural loop [41]. A functional magnetic resonance imaging investigation demonstrated that acupuncture at “Fengchi” (GB 20) specifically targeting the cerebello–thalamo–cortical (CTC) circuit to alleviate the PD tremor [42]. Acupuncture may also alleviate autism symptoms through activating endogenous OT signals by stimulating specific neural circuits in the brain rather than affecting the entire brain with pharmacotherapy [28]. The clinical studies from Beijing Wucailu Autism Rehabilitation Center provide solid evidences that children in the TEAS group had better scores in Childhood Autism Rating Scale (CARS) as well as in the Autism Behavior Checklist (ABC) compared with the ASD children who only received rehabilitation training, and the levels of AVP and OT in plasma were significantly higher than those in the control group [43].

## 4 Oxytocin stimulates anandamide release in the nucleus accumbens

The endocannabinoids system (ECS) is an essential neuro-modulatory system that involves the regulation of neurotransmission and synaptic plasticity in a broad range of social emotional responses and cognitive functions. ECS consists of two primary cannabinoid receptors (CB1 and CB2), their endogenous lipid ligands, endocannabinoids, including *N*-arachidonoylethanolamine (anandamide, AEA), 2-arachidonoylglycerol (2-AG)), and their biosynthesis- and degradation-related enzymes. ECS has been shown to be implicated in social behavior regulation [44]. Recently, dysregulation of endocannabinoid signaling has been confirmed in both genetic and epigenetic models of ASD [45,46]. Pharmacological blockade of fatty acid amide hydrolase (FAAH), the main degrading enzyme of AEA, rescued ASD behavioral defects and comorbidities in different preclinical models [47]. Cannabidiol (CBD), the major non-psychoactive phytocannabinoid in cannabis is effective in improving the symptoms of ASD [48].

With respect to OT and ECS, rodent studies have suggested a complex relationship between CB1 and OT, particularly in social behavior. Chemogenetic activation of PVN OT neurons increased nucleus accumbens (NAc) endocannabinoid AEA content in an OTR-dependent manner, suggesting that the artificial activation of PVN OT neurons induced local AEA release [49]. These studies indicate that deficits in the AEA signaling mechanism driven by OT may contribute to social impairment in ASD and a correction of such deficits may provide a novel strategy for the treatment of ASD-related social impairments.

Our previous work and other animal studies have consistently shown that 2 Hz EA can increase endogenous cannabinoid AEA levels in both the peripheral skin tissues and the central nervous system [50,51]. These findings support the hypothesis that acupuncture can, on the one hand, directly up-regulate the signal of endogenous cannabinoid system to promote social interaction, and, on the other hand, by regulating OT signals to treat the behavioral symptoms of autism, it can also indirectly up-regulate the level of endogenous cannabinoid.

## 5 Serotonin and dopamine are involved in acupuncture-mediated oxytocin rewarding

Both clinical and preclinical studies have confirmed the role of serotonin in the pathophysiology of autism. In addition, the imbalance of dopamine (DA) in specific brain regions may contribute autistic behaviors. These studies suggested that ASD could be closely connected to serotoninergic and dopaminergic systems [52,53].

Animal studies have shown that OT acts on serotoninergic and dopaminergic neurons and participates in many forms of social behaviors [54]. Therefore, it is conceivable that the beneficial characteristics of social interaction can be mediated by the coordinated activity of OT and serotonin (5-HT) in NAc. Walsh et al. have shown that optogenetic stimulating 5-HT release in the NAc or pharmacological activation of NAc 5-HT1b receptors rescues social deficits in 16p11.2 deletion autism mouse model [55]. In addition, autistic children have shown defects in the mesocorticolimbic dopaminergic signaling pathway, such as decreased prefrontal cortex dopamine release [56] and reduced NAc neural response [57]. PVN OT neurons project directly to the ventral tegmental area (VTA) and activate OTRs to regulate the social behavior. Consistent with OT-driven increased activity of VTA DA neurons, VTA OT administration increases DA release as well as sociability, thereby increasing DA release in the NAc, and activating PVN-VTA circuit enhances prosocial behavior in murine models [58]. It is known that EA at different frequencies can promote the release of different neurotransmitters [35]. Acupuncture at the specific acupoint (“Shenshu” (BL23)) increased NAc 5-HT release in rats [59]. Whereas 2 Hz EA in another region (“Zusanli” (ST 36)) was able to elevate both DA and 5-HT level in the rat hypothalamus [60].

These findings indicate that acupuncture may also promote social interaction by directly regulating the 5-HT or DA system, or by facilitating the release of neurotransmitters in the brain’s rewarding region through the oxytocinergic systems.

## 6 Conclusion

**Figure 1:**
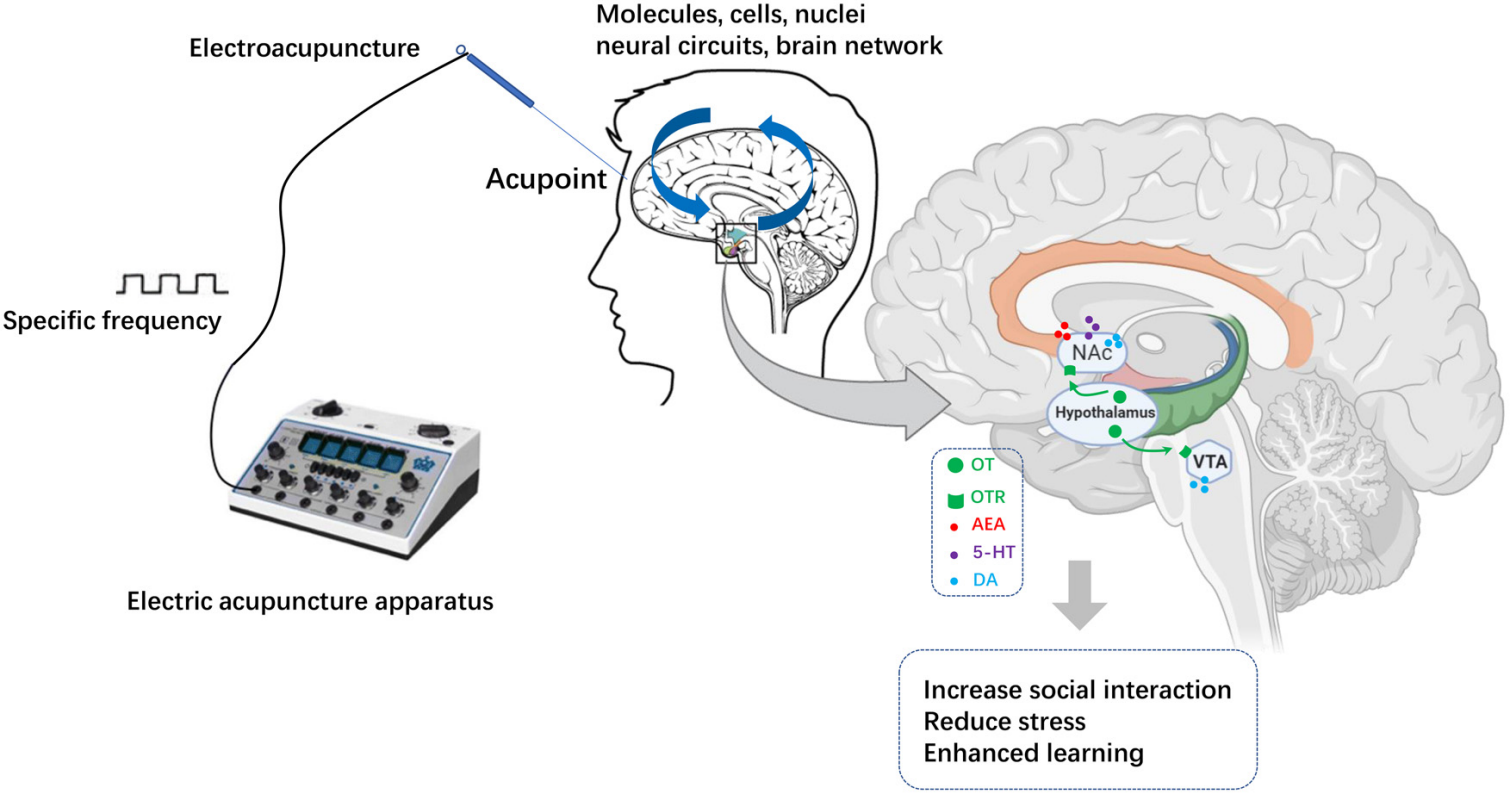
A hypothetical model of the possible mechanism of acupuncture for the treatment of autism. Acupuncture or electric acupuncture (EA) at specific acupoints with a certain frequency alleviates impaired social behaviors by directly up-regulating the oxytocin level in the hypothalamus or implicitly activating the endogenous cannabinoid, 5-HT, or DA signal pathway. OT: oxytocin; OTR: oxytocin receptor; AEA: anandamide; NAc: nucleus accumbens; VTA: Ventral tegmental area.

In reviewing previous and current acupuncture and ASD studies, we speculate that acupuncture may improve the behavioral symptoms of autism by directly up-regulating the level of OT in the hypothalamus and perhaps further promoting the synthesis, recruitment, and release of endogenous cannabinoid AEA, 5-HT, DA by increasing the level of OT in the key regions of the regulatory reward system in the brain (Figure 1). Future studies will uncover the underlying mechanisms of the treatment effects of acupuncture on ASD through applying neural tracing, brain mapping, calcium imaging, optogenetics, chemogenetics, and *in vivo* multichannel recording techniques. Human studies rely primarily on peripheral plasma OT measurement, but the correlation between central and peripheral OT has not been understood. In autistic children, plasma OT levels are lower than in age-matched controls, but many aspects of the metabolic pathways of OT are still poorly understood. It is also necessary to study whether there is insufficient use of OT in autistic children in the future. Currently, most studies are still in the preclinical stage on the relationship between ASD and OT. A thorough investigation of how acupuncture activates endogenous OT signals through affecting specific neural circuits to treat autism will be a critical step in bridging the translational gap between animal models and human therapeutics.
